# Supplementary material for: Structure of amyloid-β (20-34) with Alzheimer’s-associated isomerization at Asp23 reveals a distinct protofilament interface
Source: Nat Commun. 2019 Jul 26;10:3357. doi: 10.1038/s41467-019-11183-z (PMC6659688; doi:10.1038/s41467-019-11183-z)
Supplement: Supplementary file 1 — Supplementary Information [file 41467_2019_11183_MOESM1_ESM.pdf]

Supplementary Information

Structure of amyloid-β (20-34) with Alzheimer's-associated isomerization at Asp23 reveals a distinct protofilament interface

R.A. Warmack *et al.*

Supplementary Methods

Determination of L-isoaspartate levels by the PCMT1 methanol vapor diffusion assay

PCMT1 was used as an analytical reagent to quantify L-isoAsp levels in $A\beta^{20\text{-}34}$ peptide solutions or aggregates. Aggregates of $A\beta^{20\text{-}34}$ or $A\beta^{20\text{-}34,\ isoAsp23}$ were formed at 3.2 or 1.6 mM, respectively in 50 mM Tris-HCl, pH 7.5, 150 mM NaCl with 1% DMSO. In a final volume of 100 µL, 130 pmol of either these aggregates or freshly dissolved, filtered peptide solutions were incubated for 2 h at 37 °C with 5 µg PCMT1 (purified as a His-tagged enzyme from *Escherichia coli (E. coli)* containing the expression plasmid #34852 available from Addgene.com as described by Patananan et al., 2014[1] with a specific activity at 37 °C of 5,300 pmol of methyl esters formed on KASA(isoD)LAKY/min/mg of enzyme). Final concentrations in the reactions included 135 mM Bis-Tris-HCl, pH 6.4, and 10 µM S-adenosyl-l-[methyl3H]methionine ([3H]AdoMet) (prepared by a 1600-fold isotopic dilution of a stock of 72 Ci/mmol [3H]AdoMet (PerkinElmer Life Sciences, NET155H00) with nonisotopically labeled AdoMet (p-toluenesulfonate salt; Sigma-Aldrich A2408)). The reaction was stopped by adding 10 µL of 2 M sodium hydroxide, and 100 µL of the 110 µL mixture was transferred to a 9 by 2.5 cm piece of folded thick filter paper (Bio-Rad; catalog number 1650962) wedged in the neck of a 20-mL scintillation vial above 5 mL scintillation reagent (Safety Solve, Research Products International, catalog number 121000), tightly capped, and incubated at room temperature. After 2 h, the folded filter papers were removed, the caps replaced, and the vials were counted thrice for 5 minutes each in a Beckman LS6500 scintillation counter. Background radioactivity in a reaction containing no substrate was determined by incubating the recombinant human PCMT1, 135 mM Bis-Tris-HCl buffer, and 10 µM [3H] AdoMet as described above and was subtracted from the value obtained in experimental samples. Samples were analyzed in triplicate.

Fibril formation of full length Aβ 1-40

Wild-type and L-isoAsp23 Aβ 1- 40 were resuspended at a final concentration of 40 μM with 10 μM ThT in 10 mM phosphate, 127 mM NaCl, and 2.7 mM KCl, pH 7.4 (PBS). Fibrils were formed at 37 °C with continuous shaking at 600 rpm in a Varioskan plate reader. Fluorescence of three replicate wells was monitored in a 96 well plate, with readings taken every 5 min (excitation – 440 nm, emission - 482 nm, bottom read). Concentrations of all peptide solutions were verified by absorbance at 280 nm. EM images were recorded on an FEI Tecnai G_2 TF20 TEM.

Synthesis and purification of Aβ 1-40

Peptide syntheses were carried out at 0.1 mmol scale. A 2-chlorotrityl chloride resin (Advanced Chemtech; SC5055) was selected as the solid support with a nominal loading of 1.0 mmol/g. Each loading of the first amino acid was executed by adding 0.1 mmol (0.34 mg) of Fmoc-Val-OH and 0.4 mmol (70 μL) of diisopropylethylamine (DIPEA), dissolved in 10 mL of dichloromethane (DCM), to 0.5 grams of resin. This mixture was gently agitated by bubbling with air. After 30 minutes, the supernatant was drained, and the resin was rinsed twice with 15 mL aliquots of capping solution, consisting of 17:2:1 DCM/MeOH/DIPEA. With the first amino acid loaded, the elongation of each polypeptide was completed in a CEM Liberty Blue^{TM} Microwave Peptide Synthesizer.

A 1.0 M solution of N,N'-diisopropylcarbodiimide (DIC; Advanced Chemtech; RC8120/33084) in DMF was used as the primary activator, and a 1.0 M solution of ethyl cyanohydroxyiminoacetate (oxyma; CEM; S001-C/CEM1802117001-052118) in DMF, buffered by 0.1 M of DIPEA was used as a coupling additive. All protected, natural amino acids used during syntheses were purchased from Advanced Chemtech. The following table indicates the batch numbers and the listed HPLC purities provided by the manufacturer. All amino acids from Advanced Chemtech contain less than 0.5% of their corresponding D enantiomer, confirmed by chiral HPLC. For the synthesis of amyloid β-protein (1-40) IsoAsp23, Fmoc-Asp-OtBu (Combi-

Blocks; SS 0525/B15012; 98% purity) was used in place of the Fmoc-Asp(OtBu)-OH for position 23. The microwave synthesizer utilizes 0.2 M solutions of each amino acid.

For the deprotection of N-termini, Fmoc protecting groups, a 9% w/v solution of piperazine in 9:1 N-Methyl-2-Pyrrolidone to EtOH, buffered with 0.1 M of oxyma was used. For 0.1 mmol deprotection reactions, 4 mL of the above deprotection solution was added to the resin. The mixture was then heated to 90°C for 2 minutes while bubbled with nitrogen gas. The solution was drained, and the resin washed with 4 times with 4 mL aliquots of DMF. For 0.1 mmol couplings, 2.5 mL of 0.2 M amino acid solution (0.5 mmol) was added to the resin along with 1 mL of the DIC solution (1.0 mmol) and 0.5 mL of oxyma solution (0.5 mmol). This mixture was agitated by bubbling for 2 minutes at 25°C and then heated to 50°C followed by 8 minutes of bubbling. After the coupling reaction, an Fmoc deprotection reaction without conducting additional washes. Double couplings were used to ensure complete coupling, starting from the attachment of Ala21.

After the last deprotection, the resins were washed with methanol, diethyl ether, dried over vacuum, and introduced to a cleavage cocktail consisting of: 20 mL of trifluoroacetic acid (TFA); 0.330 mL of 1,2-ethanedithiol (EDT); 0.380 mL of water; 0.225 grams of ammonium iodide (NH_4I); 0.300 mL of dimethyl sulfide (DMS); 0.150 mL of triisopropylsilane (TIS). After 2 hours of vigorous stirring, the mixtures were filtered, and the filtrate concentrated in vacuo. The residues were triturated with cold diethyl ether, and 0.412 g and 0.444 g of precipitated, crude peptides were collected by filtration for amyloid β-protein (1-40) WT and Amyloid β-Protein (1-40) IsoAsp23, respectively.

The purity of the crude product, amyloid β-protein (1-40) WT, was analyzed by RP-HPLC, using a Hewlett Packard 1090 Series II Liquid Chromatography System equipped with a Kinetex (Phenomenex, C18, 5 μm, 100 Å, 4.6 x 250 mm) column. Ultrapure water with 0.1% TFA, and a 1:9 water to acetonitrile solution with 0.095% TFA were selected as mobile phases [A] and [B], respectively. The flow rate was set at 1.0 mL/min and the gradient used is detailed

in **Supplementary Table 2**. The UV absorption at 214 nm was monitored. The resulting chromatogram is shown in **Supplementary Figure 6**.

The crude peptide was then purified by RP-HPLC, using an Interchim puriFlash® 4125 Preparative Liquid Chromatography System equipped with a Luna (Phenomenex, C18(2), 5 µm, 100 Å, 30 x 100 mm) column. For purification, two buffer systems were utilized. Initial purifications and salt exchanges were executed with a 1:99 acetonitrile to water solution, buffered by 15 mM NH_4OH ([A]) and a 2:3 water to acetonitrile solution, buffered by 15 mM of NH_4OH ([B]). For better resolution of diastereomers and other impurities, ultrapure water, buffered by 14 mM of $HClO_4$, and a 2:3 water to acetonitrile solution, buffered by 5.6 mM of $HClO_4$, were selected as mobile phases A and B, respectively. Buffer systems with TFA were avoided because large decreases in peak efficiency were observed for the product peak in such systems, precluding compound recovery. The purification gradients used are described in **Supplementary Table 3**. Many purifications were truncated after the peak was eluted in order to prepare the LC instrument immediately for the next injection, minimizing the oxidation of methionine. UV absorptions at 214 nm was monitored. 62 mg of crude peptide was dissolved in 10 mL of an aqueous 1.5 M NH_4OH solution and loaded onto the column via valve injection. **Supplementary Table 3** displays the details of the gradient and **Supplementary Figures 7** and **8** show details of the corresponding HPLC trace.

The LC system has been modified (customization provided by Interchim) to allow for direct loading of solutions onto the column through the pump (see **Supplementary Figure 9**). The fractions collected from Purification 1 were subjected to a two-fold dilution and loaded directly back onto the column for purification. **Supplementary Table 3** displays the details of the gradient and **Supplementary Figures 10** and **11** shows details of the corresponding HPLC trace.

The collected fractions from Purification 2 were pooled and directly loaded back onto the column. The peptide was subjected to a salt exchange, displacing the perchlorate ions with

ammonium ions. The resulting fractions were pooled, flash frozen, and lyophilized. The lyophilized powder was dissolved in 10 mL of an aqueous 1.5 M NH_4OH solution, loaded onto the column via valve injection, and subjected to a third purification. **Supplementary Table 3** displays the details of the gradient and **Supplementary Figures 12** and **13** show details of the corresponding HPLC trace.

The collected fractions from Purification 3 were pooled and directly loaded back onto the column. The peptide was subjected to a salt exchange, displacing the perchlorate ions with ammonium ions. The resulting fractions were flash frozen separately and lyophilized. The purity of these fractions was analyzed by RP-HPLC, using a Hewlett Packard 1090 Series II Liquid Chromatography System equipped with a Kinetex (Phenomenex, C18, 5 µm, 100 Å, 4.6 x 250 mm) column. Ultrapure water, buffered by 14 mM of $HClO_4$, and a 1:9 water to acetonitrile solution buffered by 1.4 mM of $HClO_4$, were selected as mobile phases A and B, respectively. The flow rate was set at 1.0 mL/min and the gradient used is detailed in **Supplementary Table 2**. The UV absorption at 214 nm was monitored, and the peaks in the resulting chromatogram were manually integrated.

The purest lyophilized fraction yielded 0.3 mg of material from 62 mg of the crude peptide. To obtain more Aβ (1-40) WT, the purification was repeated with 87 mg of crude material, producing 5.3 mg of material with similar purity. The increase in yield was due to better fraction selection, accounting for solvent delay between the flow cell in the UV detector and the fraction collector. **Supplementary Figures 14** and **15** show the purity analysis via HPLC of the second purification, indicating an estimated purity of 93% for the purest fraction.

The purified Aβ (1-40) WT was also characterized by ESI-MS (**Supplementary Figures 16** and **17**) via direct injection into a Q-Exactive™ Plus Hybrid Quadrupole-Orbitrap™ Mass Spectrometer. The calculated average mass for $C_{194}H_{295}N_{53}O_{58}S$: 4327.148 g/mol, m/z calculated: $[M+3H]^{3+}$ = 1443.39; $[M+4H]^{4+}$ = 1082.79; $[M+5H]^{5+}$ = 866.44; $[M+6H]^{6+}$ = 722.20. Observed: 1443.3913; 1082.7955; 866.4374; 722.1991.

The purity of the crude product, amyloid β-protein (1-40) IsoAsp23, was analyzed by RP-HPLC, using a Hewlett Packard 1090 Series II Liquid Chromatography System equipped with a Kinetex (Phenomenex, C18, 5 μm, 100 Å, 4.6 x 250 mm) column. Ultrapure water with 0.1% TFA, and a 1:9 water to acetonitrile solution with 0.095% TFA were selected as mobile phases [A] and [B], respectively. The flow rate was set at 1.0 mL/min and the gradient used is detailed in **Supplementary Table 2**. The UV absorption at 214 nm was monitored. The resulting chromatogram is shown in **Supplementary Figure 18**.

The crude IsoAsp23 peptide was purified by RP-HPLC with the same methods and gradients as the WT peptide. The resulting fractions were also analyzed in the same manner. The purest lyophilized fractions of Aβ (1-40) IsoAsp23 yielded 4.75 mg of material from 84 mg of crude peptide. **Supplementary Figures 19** and **20** shows the purity analysis of one such fraction via HPLC, indicating an estimated purity of 94%.

The purified Aβ (1-40) IsoAsp23 was also characterized by ESI-MS (**Supplementary Figures 21** and **22**) via direct injection into a Q-Exactive™ Plus Hybrid Quadrupole-Orbitrap™ Mass Spectrometer. The calculated average mass for $C_{194}H_{295}N_{53}O_{58}S$: 4327.148 g/mol, m/z calculated: $[M+3H]^{3+}$ = 1443.39; $[M+4H]^{4+}$ = 1082.79; $[M+5H]^{5+}$ = 866.44. Observed: 1443.3912; 1082.7959; 866.4373.

Supplementary Tables

Amino Acid	Catalog/Lot Number	Purity by HPLC
Fmoc-L-Ala-OH	FA2100/32786	99.6%
Fmoc-L-Arg(Pbf)-OH	FR2136/32914	99.6%
Fmoc-L-Asn(Trt)-OH	FN2152/32769	98.4%
Fmoc-L-Asp(tBu)-OH	FD2192/32770	99.9%
Fmoc-L-Gln(Trt)-OH	FQ2251/31862	99.6%
Fmoc-L-Glu(tBu)-OH	FE2237/32917	99.4%
Fmoc-L-Gly-OH	FG2275/32787	99.8%
Fmoc-L-His(Trt)-OH	FH2316/32530	99.1%
Fmoc-L-Ile-OH	FI2326/32125	99.9%
Fmoc-L-Leu-OH	FL2350/32771	99.7%
Fmoc-L-Lys(Boc)-OH	FK2390/32531	99.9%
Fmoc-L-Met-OH	FM2400/32127	99.7%
Fmoc-L-Phe-OH	FF2425/32484	99.7%
Fmoc-L-Ser(tBu)-OH	FS2476/32532	99.9%
Fmoc-L-Tyr(tBu)-OH	FY2563/32181	99.7%
Fmoc-L-Val-OH	FV2575/32773	99.8%

Supplementary Table 1: Catalog and lot number of amino acids purchased from Advanced Chemtech

Crude Analyses			QC Analyses		
TFA Buffer System			$HClO_4$ Buffer System		
Time (min)	[A] (%)	[B] (%)	Time (min)	[A] (%)	[B] (%)
0	95	5	0	80	20
5	80	20	5	65	35
25	60	40	25	45	55
26	0	100	26	0	100
30	0	100	30	0	100
31	95	5	31	80	20
35	95	5	35	80	20

Supplementary Table 2: Gradients utilized for purity analysis. Supplementary Figures 6 and 18 show the analyses of crude peptides, carried out in a TFA buffer system. Supplementary

Figures 14, 15, 19, and 20, show the analyses of purified peptides, carried out in a $HClO_4$ buffer system.

**Purification 1**			**Purification 2**			**Purification 3**		
**NH_4OH Buffer System**			**$HClO_4$ Buffer System**			**$HClO_4$ Buffer System**		
Time (min)	[A] (%)	[B] (%)	Time (min)	[A] (%)	[B] (%)	Time (min)	[A] (%)	[B] (%)
0	100	0	0	95	5	0	100	0
5	100	0	6	95	5	5	100	0
10	80	20	14	50	50	13	50	50
30	60	40	34	30	70	33	30	70
31	0	100	35	0	100	34	0	100
36	0	100	40	0	100	39	0	100

Supplementary Table 3: Gradients utilized for the purification of Aβ (1-40) WT and Aβ (1-40) IsoAsp23. Supplementary Figures 7 and 8 correspond to Purification 1. Supplementary Figures 10 and 11 correspond to Purification 2. Supplementary Figures 12 and 13 correspond to Purification 3.

Supplementary Figures

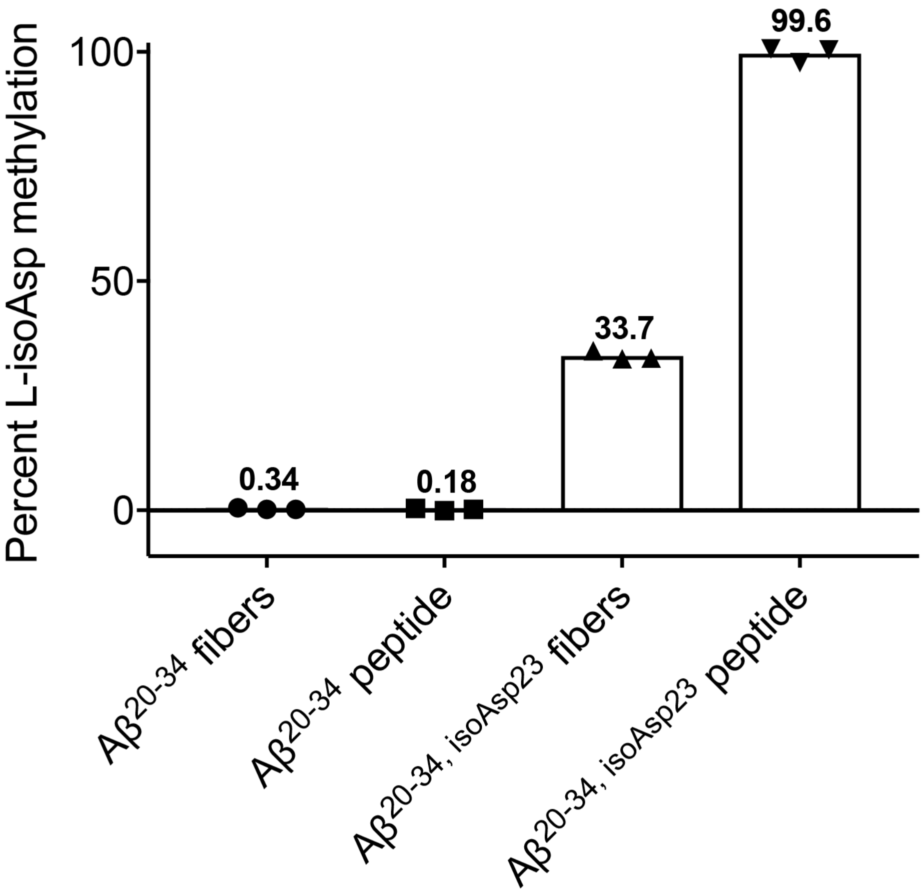

Supplementary Figure 1: PCMT1 is unable to fully methylate aggregated Aβ$^{20\text{-}34,\ isoAsp23}$. Methylation of free peptide and aggregated fibers of Aβ$^{20\text{-}34}$ and Aβ$^{20\text{-}34,\ isoAsp23}$ was detected as described in the experimental procedures. Levels of detected L-isoAsp were normalized between 0-100% methylation. The normal L-Asp Aβ$^{20\text{-}34}$ fibers and peptide were included as negative controls and were not methylated by PCMT1, as shown by the averages of 0.34% and 0.18% methylation, respectively. Source data are provided as a Source Data file.

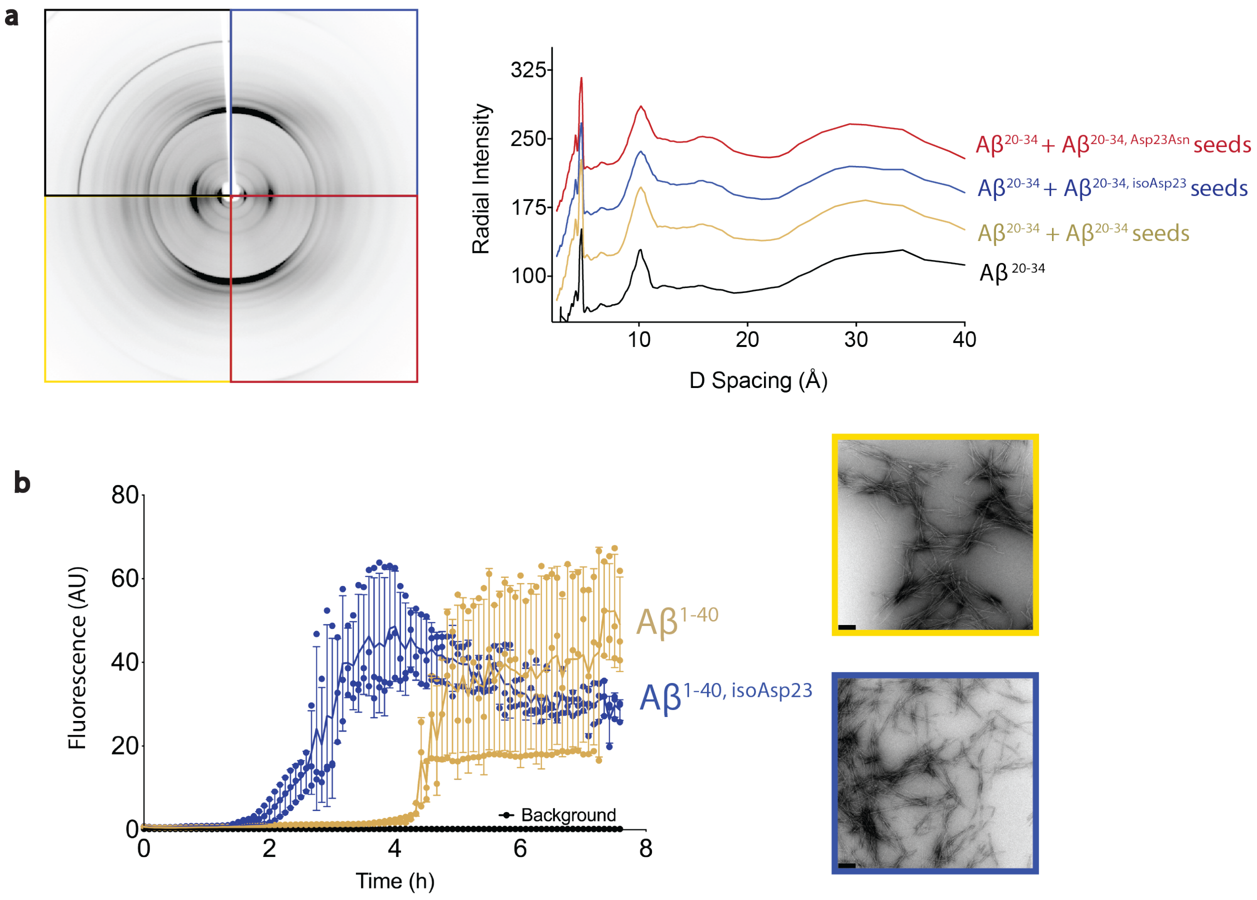

Supplementary Figure 2: Seeded Aβ$^{20-34}$ aggregates display identical fiber diffraction patterns, and the full-length Aβ$^{1-40}$ aggregation matches the Aβ$^{20-34}$ aggregation. a, Aggregates from the seeding assay of Aβ$^{20-34}$ shown in the main text Fig. 1d were ordered between glass capillaries and fiber diffraction data was collected as described in the "Methods." Radial intensity of the reflections was plotted against D Spacing (right). **b**, 20 μM wild-type Aβ$^{1-40}$ (gold lines) or Aβ$^{1-40,\ isoAsp23}$ (blue lines) were incubated at 37 °C. Fiber formation was monitored by Thioflavin T fluorescence, readings were recorded every 5 min. Each data point is shown as a round symbol, the solid line represents the mean value, and error bars represent SD of three technical replicates. A representative EM image of each condition is shown on the right, scale bars at the lower left represent 200 nm. Source data are provided as a Source Data file.

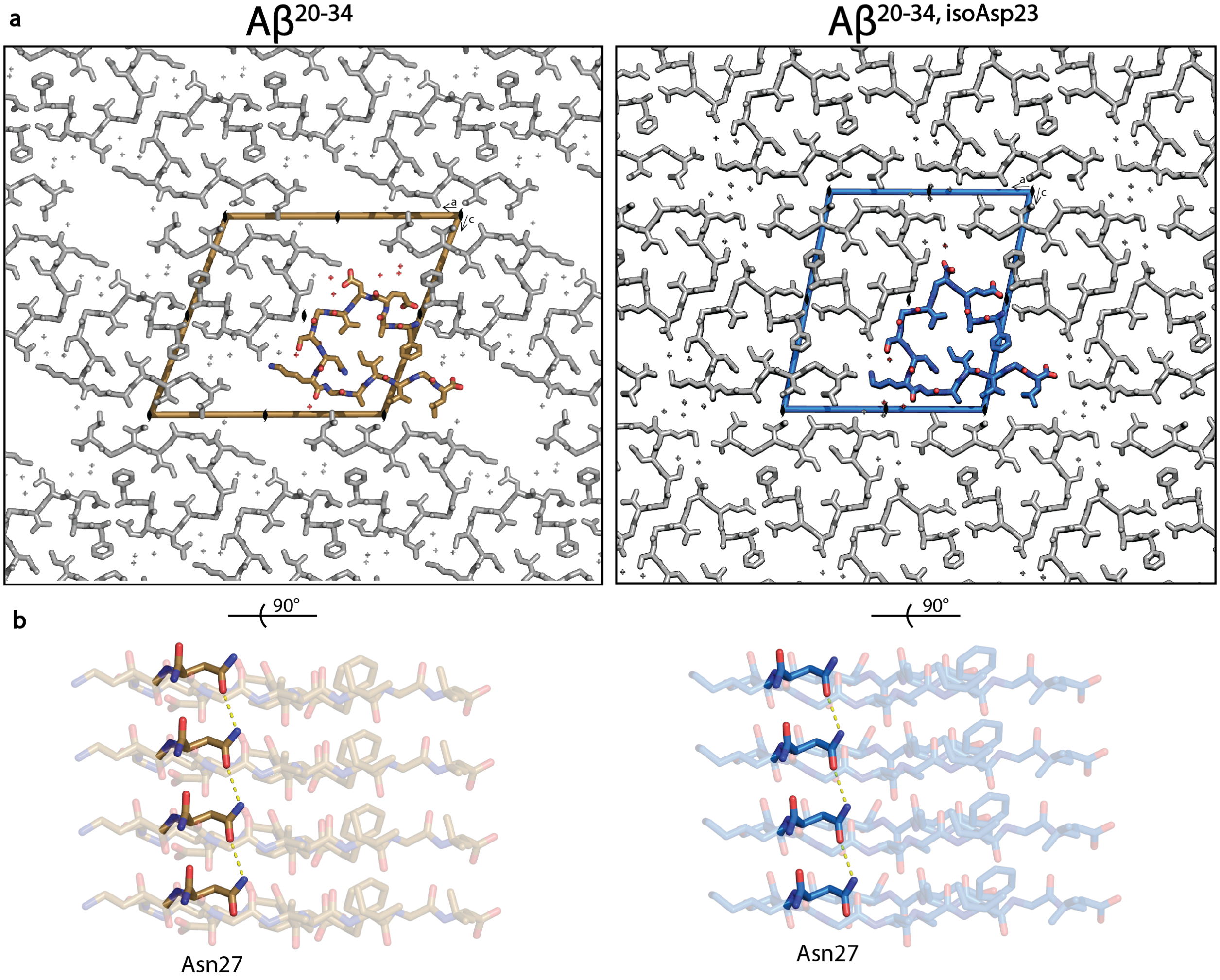

Supplementary Figure 3: A$\beta^{20\text{-}34}$ structures reveal two interfaces and a polar zipper. a, The crystal structures are shown here along the 2_1 axis of the unit cell. Black symbols (⬬) represent the 2_1 axis of symmetry. **b**, The asparagine ladder motif is shown with yellow dashed lines between strands, the structure is shown perpendicular to the protofilament axis, along the face of residues Lys28-Leu34. The dashed lines within the A$\beta^{20\text{-}34}$ structure correspond to 2.8 Å, and the dashed lines within the A$\beta^{20\text{-}34,\ isoAsp23}$ structure correspond to 2.9 Å.

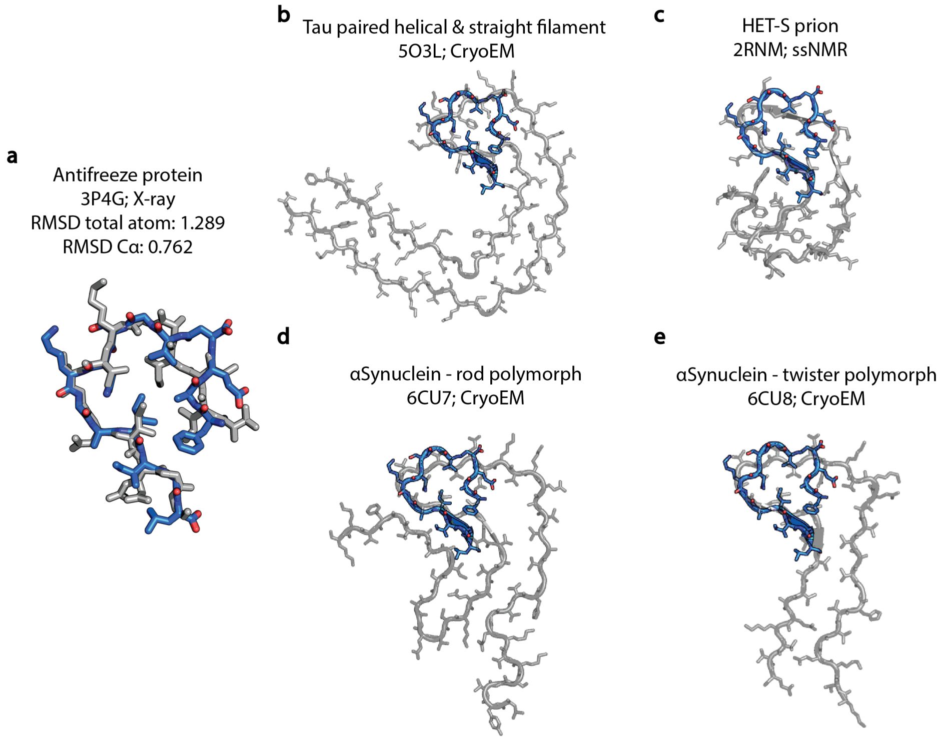

Supplementary Figure 4: A$\beta^{\mathbf{20\text{-}34,\ isoAsp23}}$ matches β-helical structures and other amyloid fibrils. **a**, The top match for the A$\beta^{20\text{-}34,\ isoAsp23}$ structure from comparison to all PDB structures via a DALI search[2] was the β-helical antifreeze protein 3P4G[3]. **b-d**, Other amyloid fibril structures, including tau[4] and αSynuclein[5] show similar tight turns (β-arches) between steric zippers.

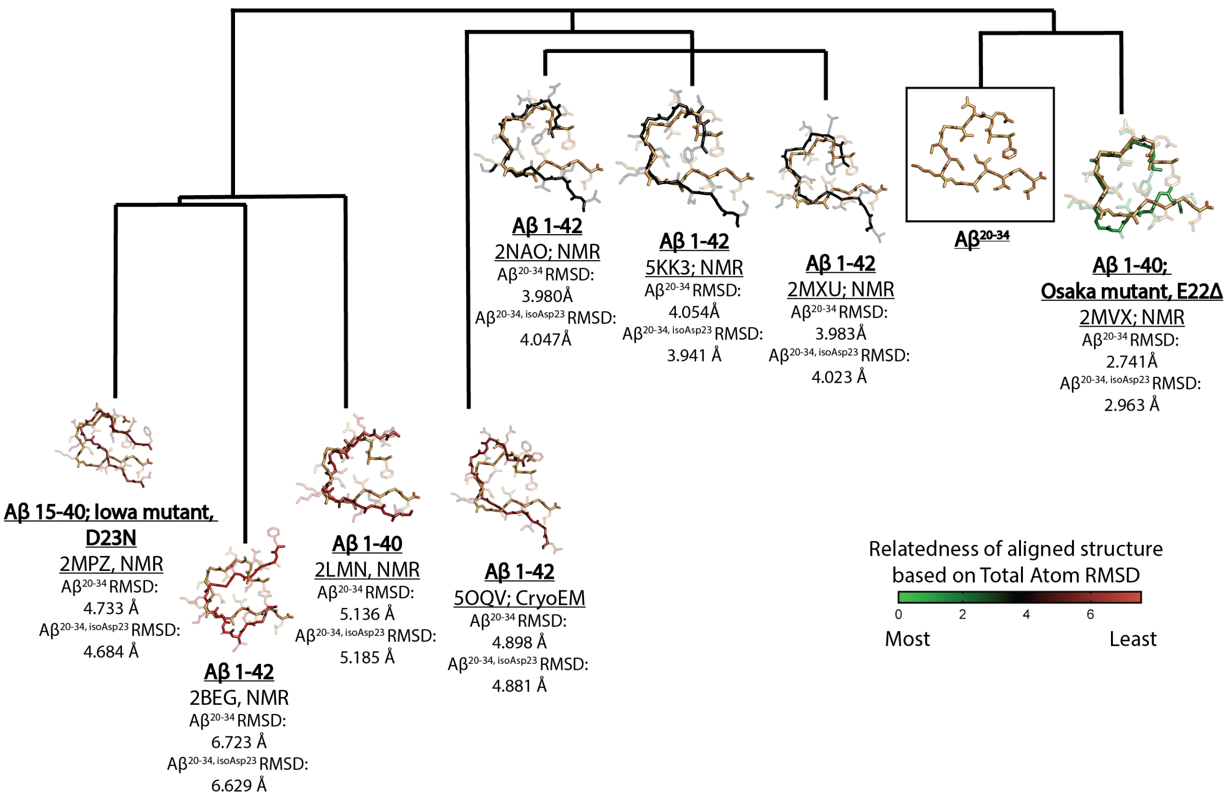

Supplementary Figure 5: Total atom RMSD relationships between Aβ structures. The $A\beta^{20\text{-}34,\ isoAsp23}$ (gold, second from right) was aligned to residues 20-34 of full-length Aβ structures[6-13]. Backbone and total atom RMSDs were calculated using CCP4. Branches of the evolutionary tree represent total atom RMSD relatedness between the structures and were generated as described in the Methods section. The backbones of the aligned structures are also colored on a scale of red to green according to how closely matched their total RMSD values are to $A\beta^{20\text{-}34}$ (legend shown on bottom right).

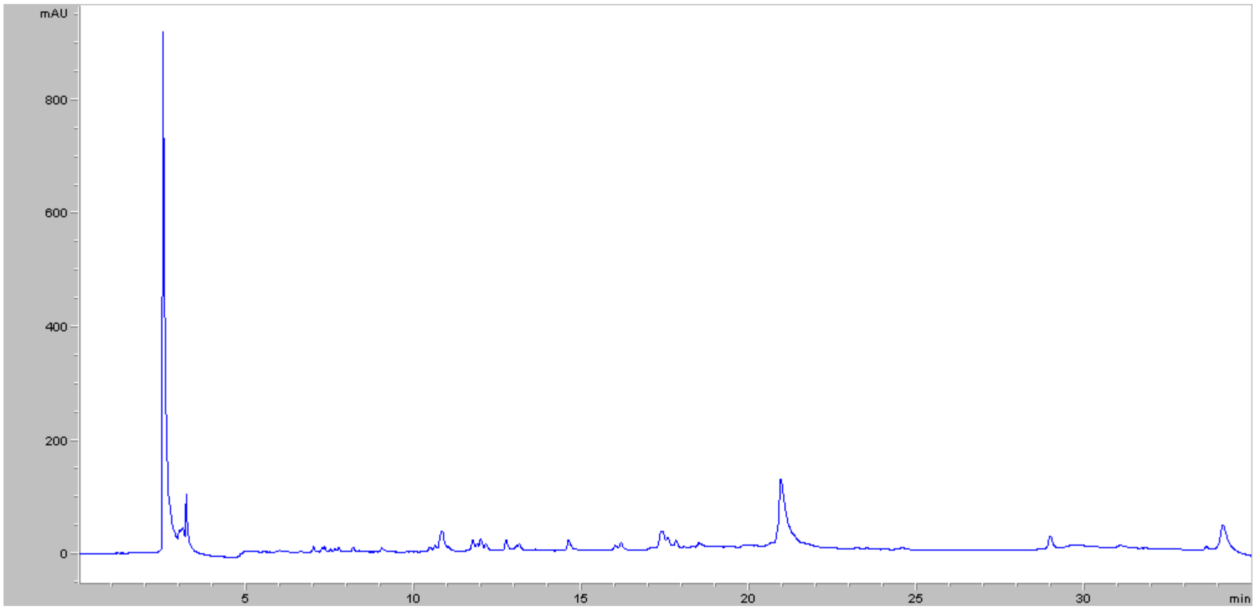

Supplementary Figure 6: Analytical HPLC trace of Aβ (1-40) WT crude. The analytes are detected by their absorbance (y-axis, mAU) at 214 nm. The gradient is 35 minutes (x-axis) long and the desired product elutes at **20.92** minutes. No significant side products were observed.

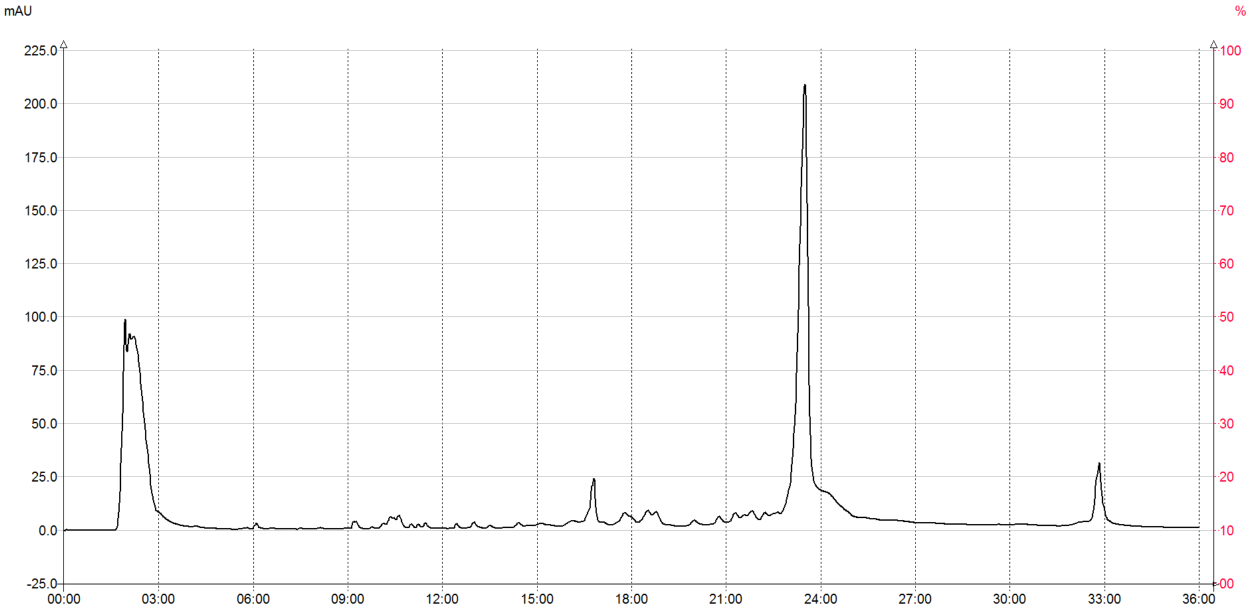

Supplementary Figure 7: HPLC trace of <u>Purification 1</u> of the Aβ (1-40) WT crude peptide using an ammonium hydroxide buffer system. Peptides are detected by their absorbance (y-axis, mAU) at 214 nm as they pass through the flow cell over time (x-axis, minutes).

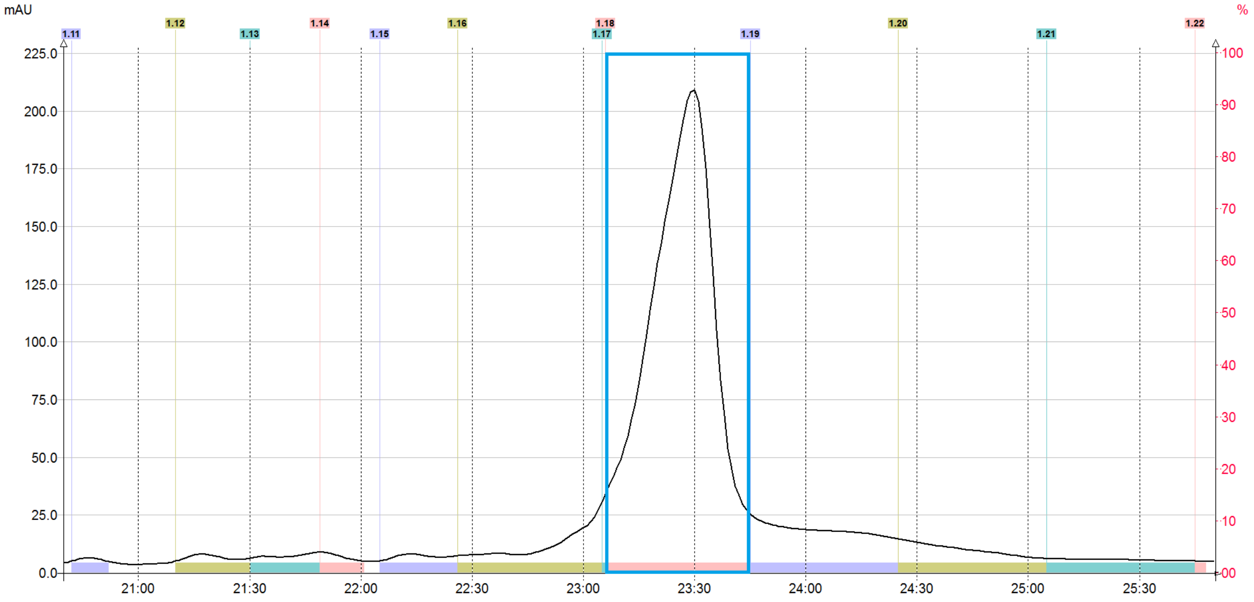

Supplementary Figure 8: Zoomed view of main Aβ (1-40) WT product peak in Supplementary Figure 7. Collected fraction(s) are indicated by the boxed region. The analytes are detected by their absorbance (y-axis, mAU) at 214 nm as they pass through the flow cell over time (x-axis, minutes).

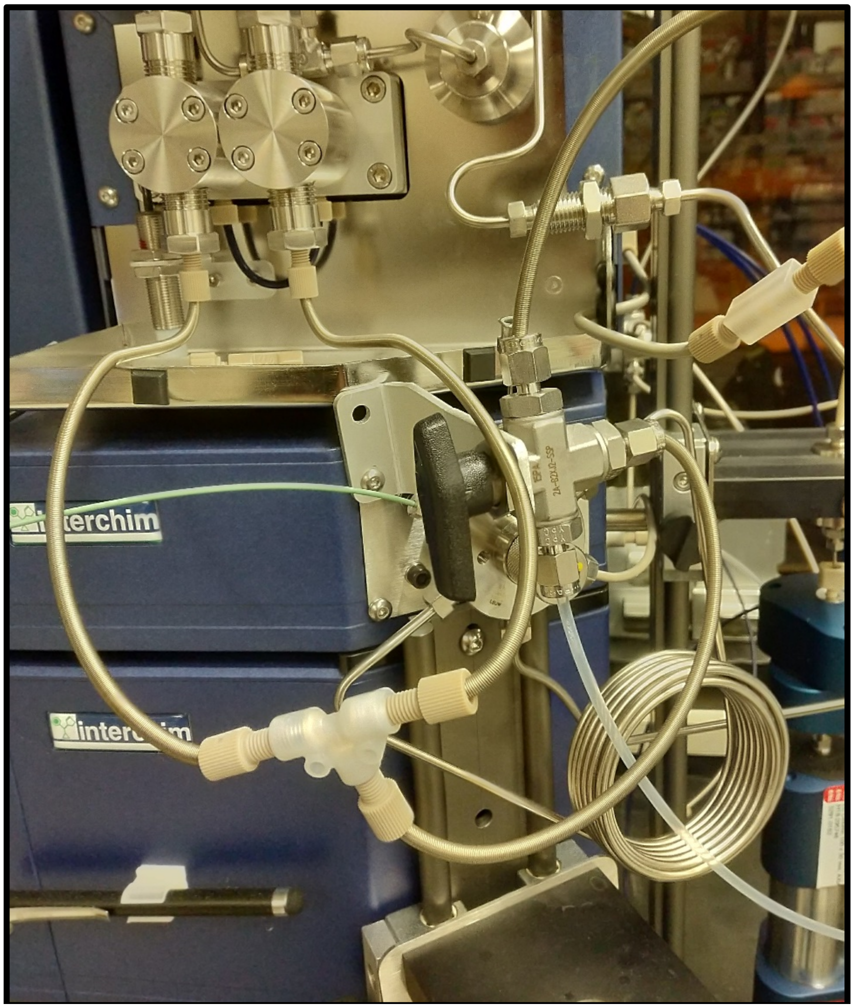

Supplementary Figure 9: Modification of Interchim puriFlash® PrepLC with an additional T-valve allows for direct loading of sample solution to column through pump.

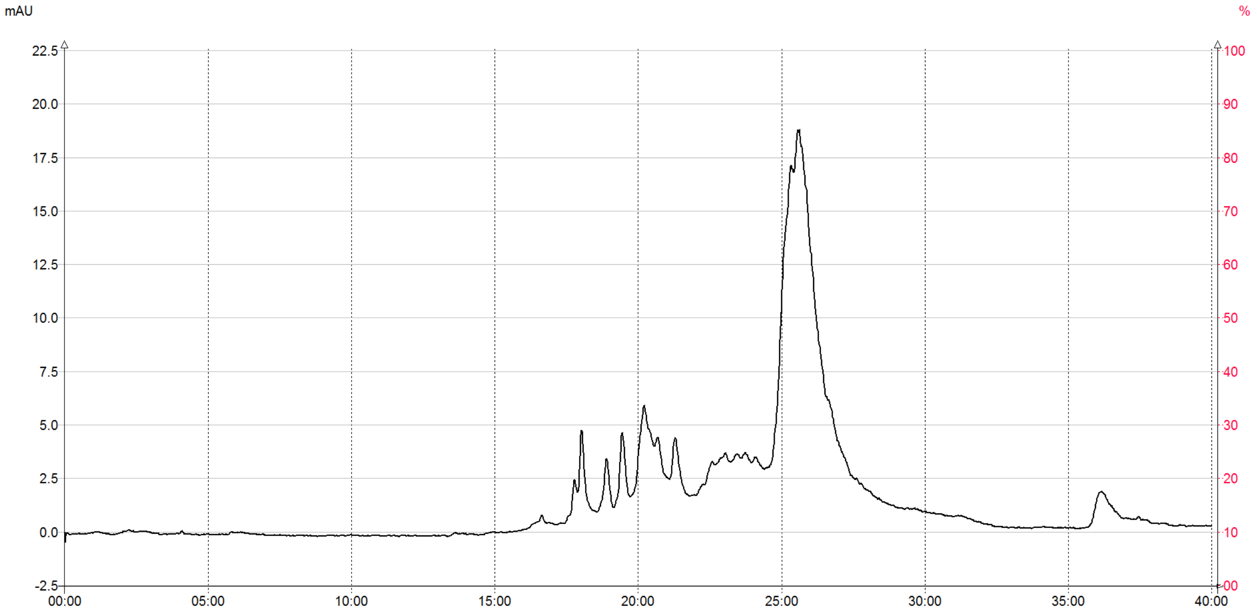

Supplementary Figure 10: HPLC trace of Purification 2 of Aβ (1-40) WT using a perchloric acid buffer system. The analytes are detected by their absorbance (y-axis, mAU) at 214 nm as they pass through the flow cell over time (x-axis, minutes).

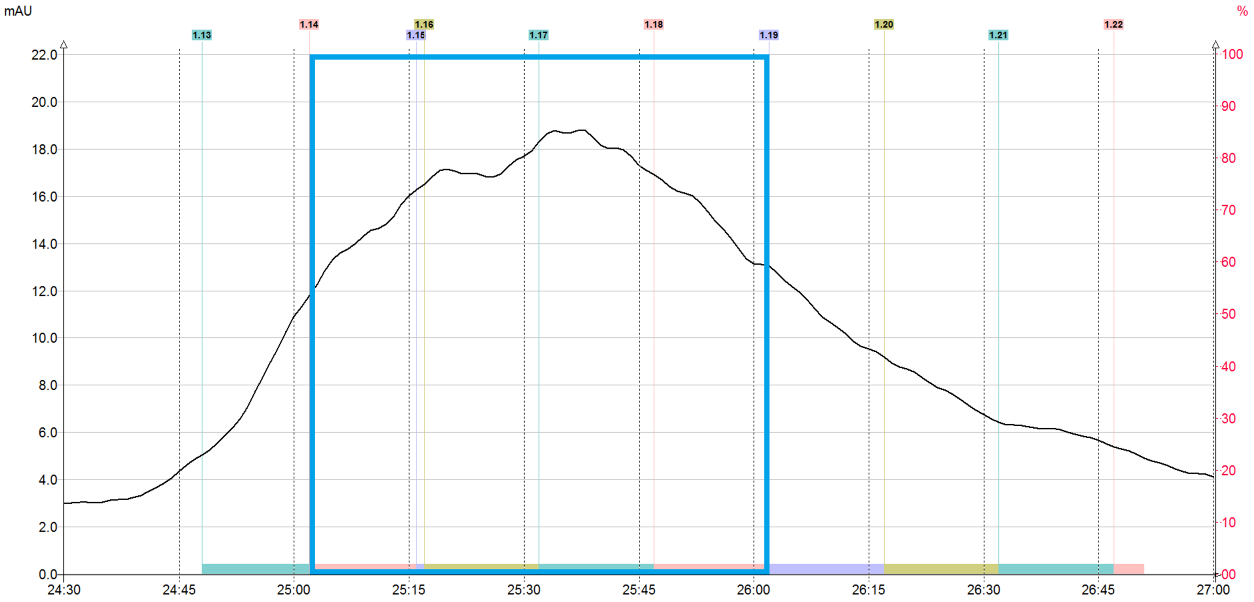

Supplementary Figure 11: Zoomed view of main Aβ (1-40) WT product peak in Supplementary Figure 10. Collected fraction(s) indicated by boxed region. The analytes are detected by their absorbance (y-axis, mAU) at 214 nm as they pass through the flow cell over time (x-axis, minutes).

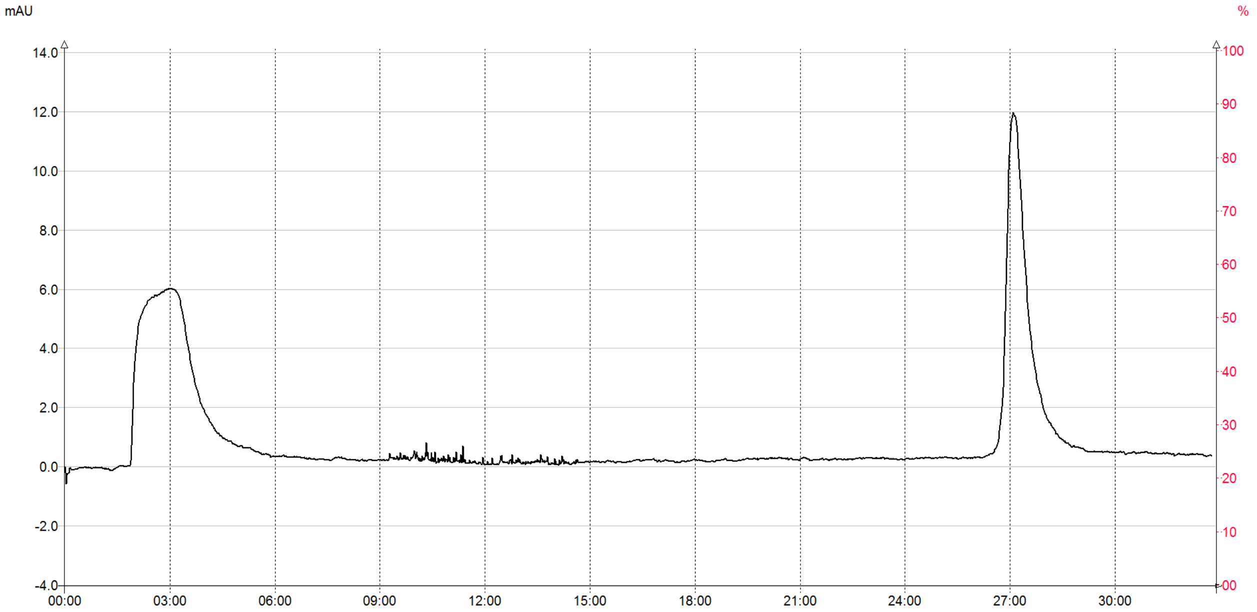

Supplementary Figure 12: HPLC trace of Purification 3 of Aβ (1-40) WT using a perchloric acid buffer system. The analytes are detected by their absorbance (y-axis, mAU) at 214 nm as they pass through the flow cell over time (x-axis, minutes).

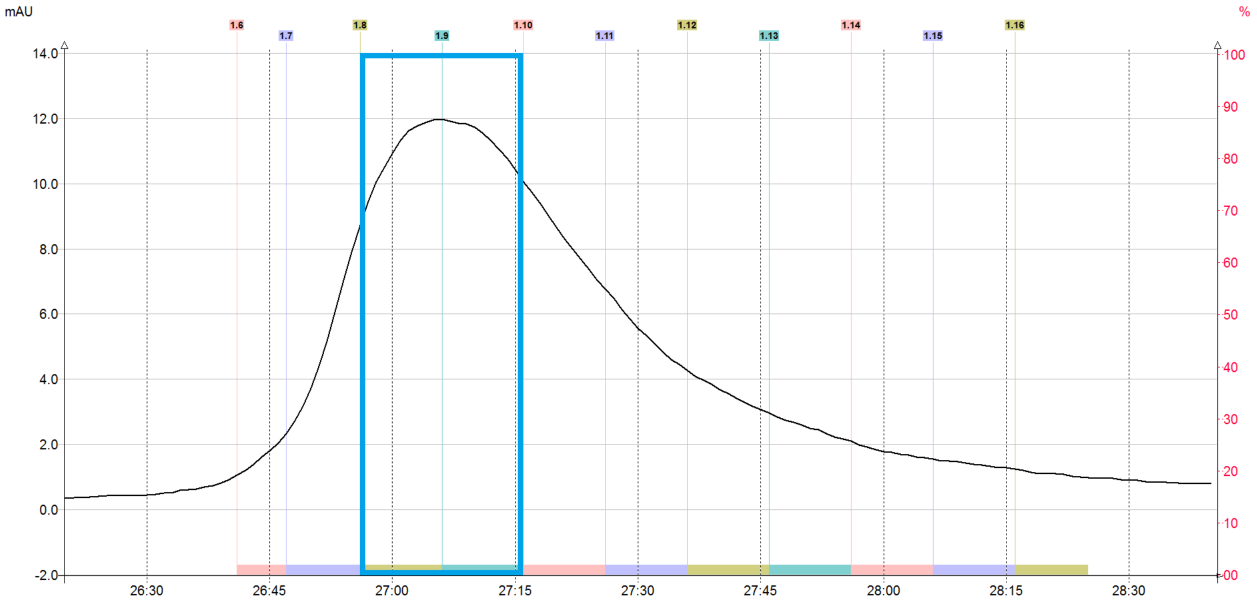

Supplementary Figure 13: Zoomed view of main Aβ (1-40) WT product peak in Supplementary Figure 12. Collected fraction(s) indicated by boxed region. The analytes are detected by their absorbance (y-axis, mAU) at 214 nm as they pass through the flow cell over time (x-axis, minutes).

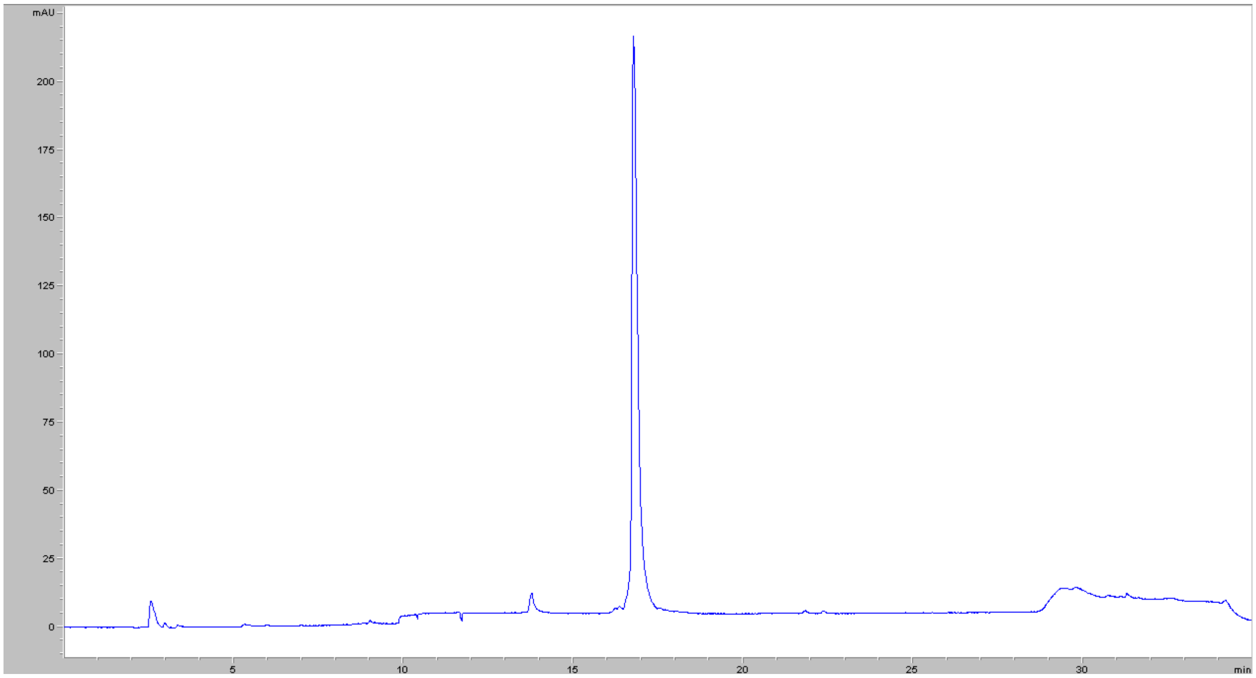

Supplementary Figure 14: Analytical HPLC trace of the purest lyophilized fraction for the second Aβ (1-40) WT purification. The analytes are detected by their absorbance (y-axis, mAU) at 214 nm as they pass through the flow cell over time (x-axis, minutes).

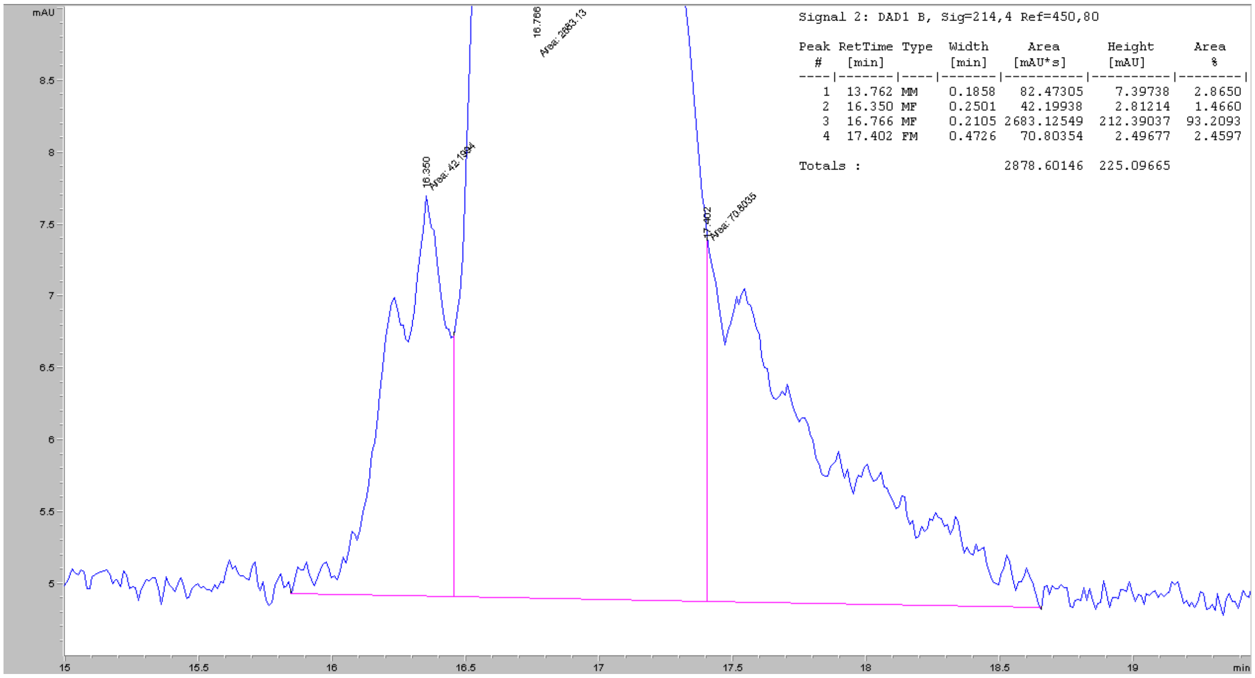

Supplementary Figure 15: Manual integration of the UV trace shown in Supplementary Figure 14 obtained by analytical HPLC. **t_R 13.76**: 82.47 mAU^2 (2.87%); **t_R 16.35**: 42.20 mAU^2 (1.47%); **t_R 16.77**: 2683.13 mAU^2 (93.20%); **t_R 17.40**: 70.80 mAU^2 (2.46%).

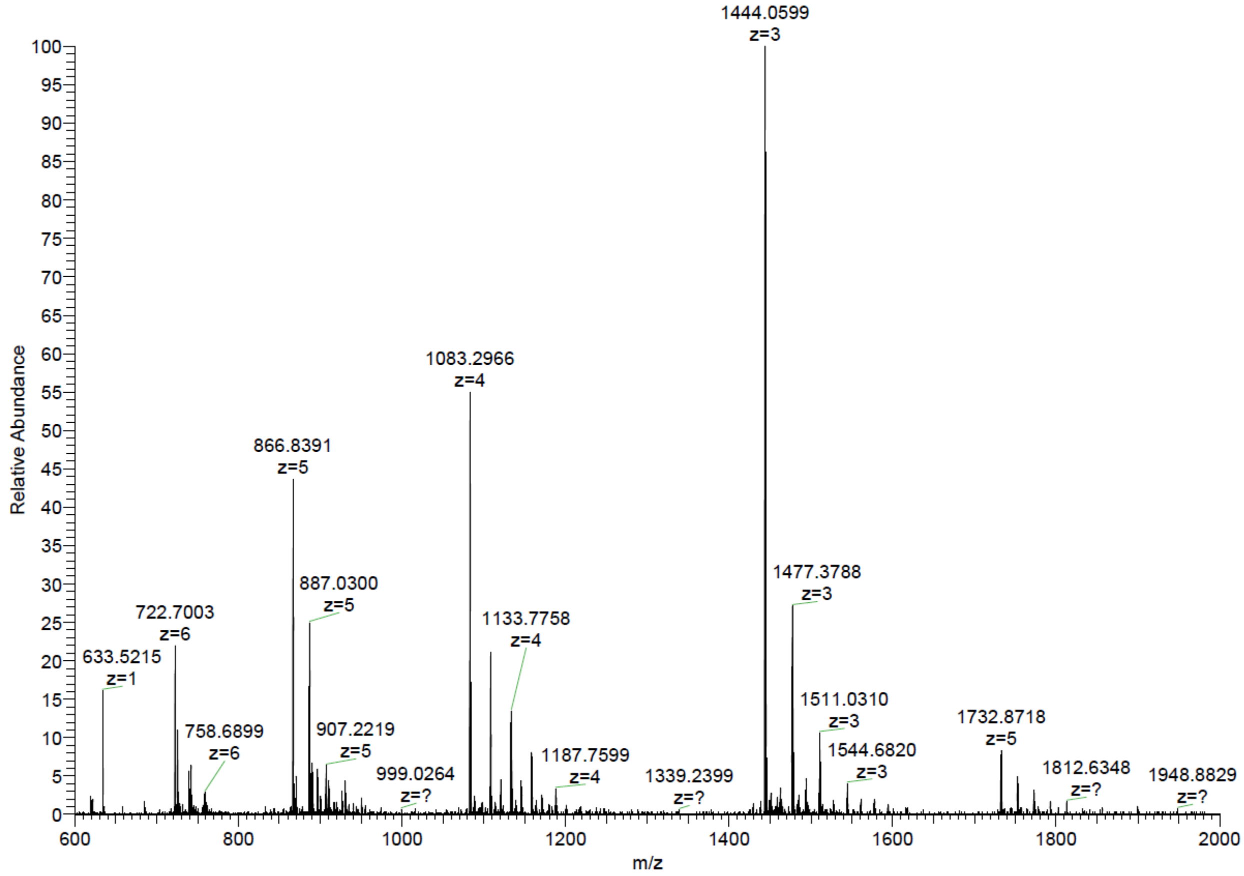

Supplementary Figure 16: Broadband mass spectrum of purified Aβ (1-40) WT collected by direct injection. The scan range was 600-2000 (m/z), and the population of each ion is represented by relative abundance.

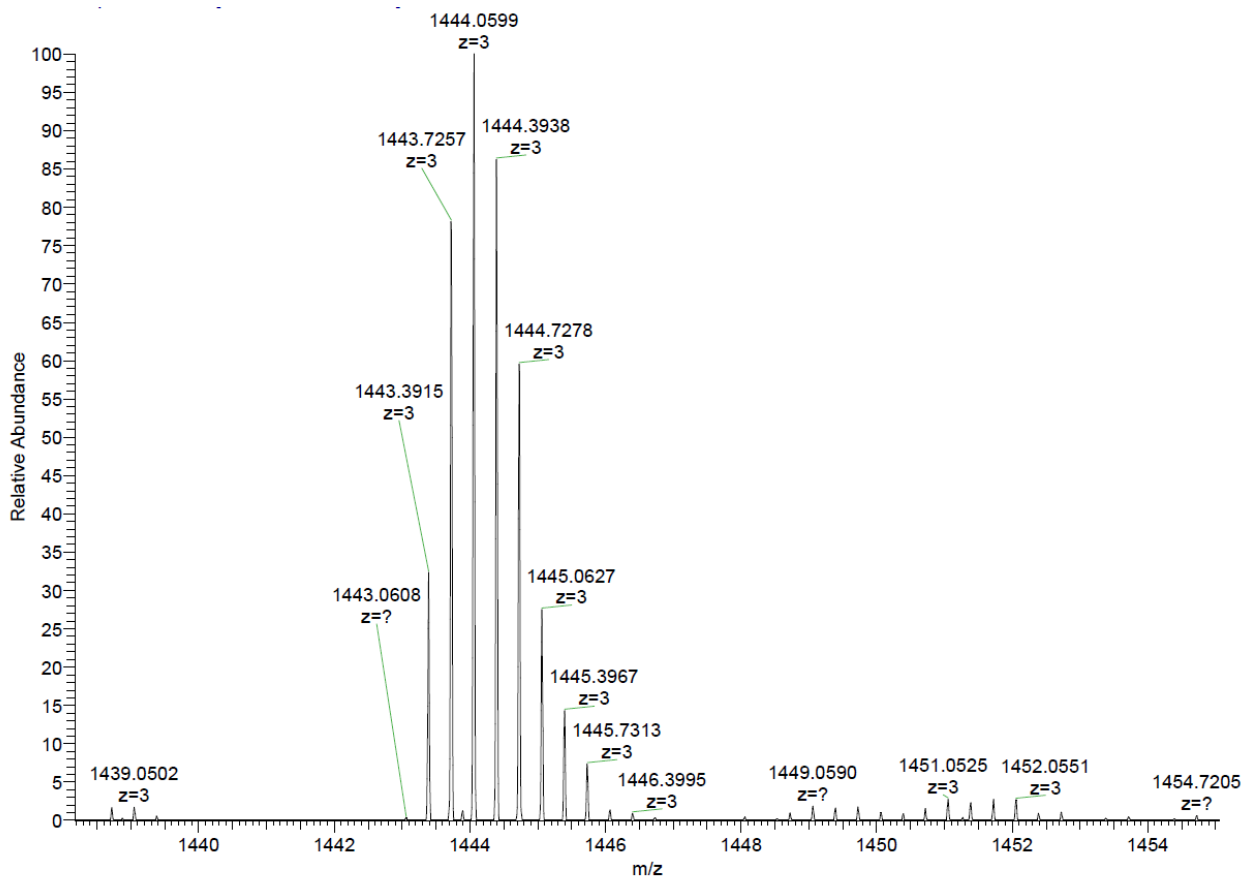

Supplementary Figure 17: Zoomed view of main Aβ (1-40) WT peak in broadband spectrum shown in Supplementary Figure 16. The monoisotopic $[M+3H]^{3+}$ is 1443.3915.

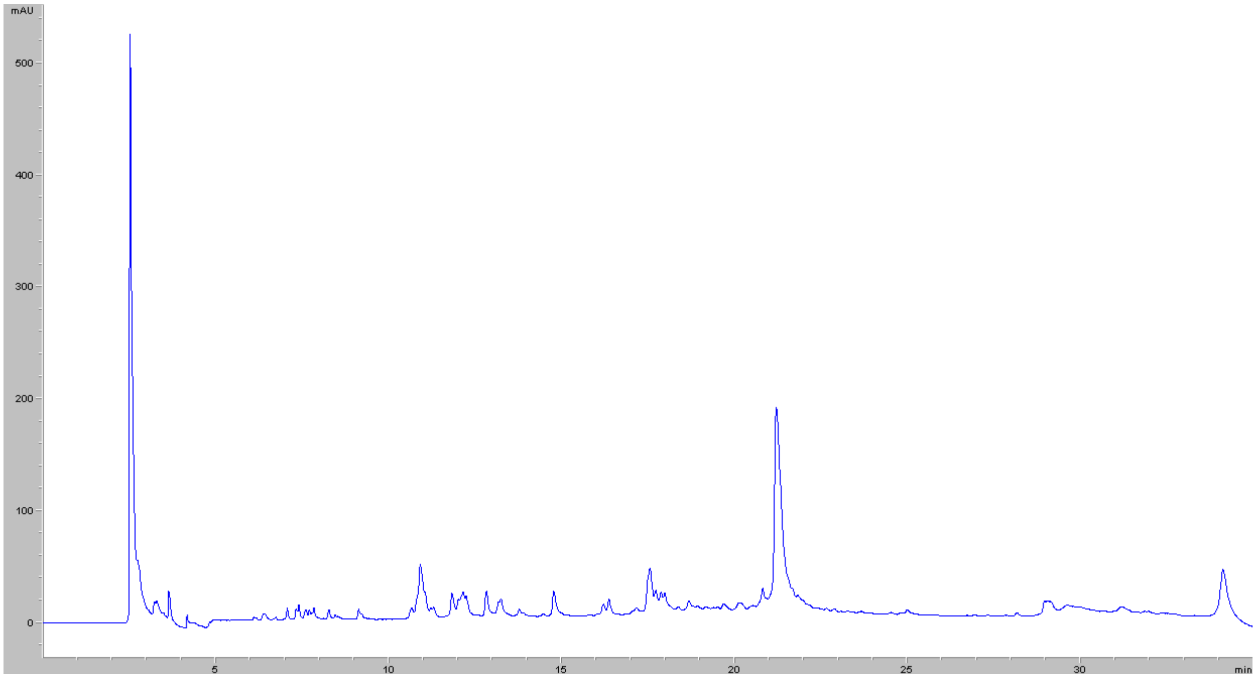

Supplementary Figure 18: Analytical HPLC trace of Aβ (1-40) IsoAsp23 crude peptide using the TFA solvent system. The analytes are detected by their absorbance (y-axis, mAU) at 214 nm. The gradient is 35 minutes (x-axis) long and the desired product elutes at **21.20** minutes. No significant side products were observed.

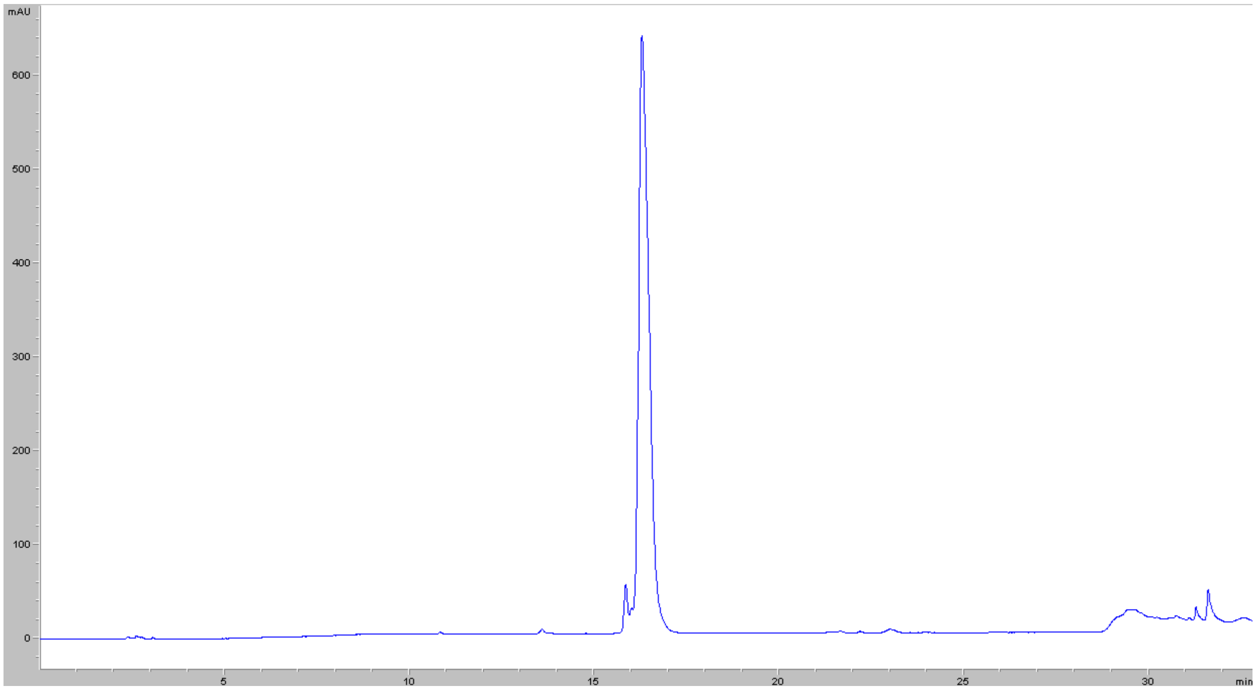

Supplementary Figure 19: Analytical HPLC trace of the purest lyophilized fraction for Aβ (1-40) IsoAsp23 from material that was subjected to the three preparative HPLC steps described in Supplementary Table 3. The analytes are detected by their absorbance (y-axis, mAU) at 214 nm as they pass through the flow cell over time (x-axis, minutes).

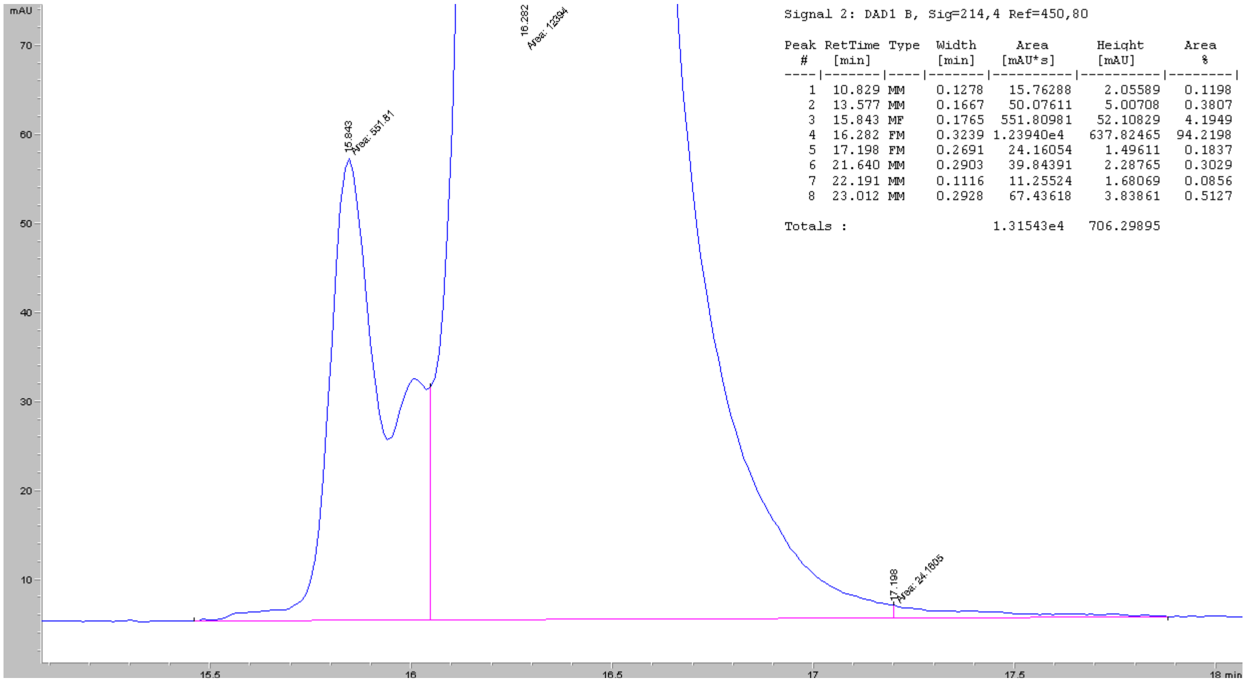

Supplementary Figure 20: Manual integration of the UV trace obtained shown in Supplementary Figure 19. **t_R 10.83: 15.76** mAU^2 (0.12%); **t_R 13.58: 50.08** mAU^2 (0.38%); **t_R 15.84: 551.81** mAU^2 (4.19%); **t_R 16.28: 12394.0** mAU^2 (94.22%); **t_R 17.20: 24.16** mAU^2 (0.18%); **t_R 21.64: 39.84** mAU^2 (0.30%); **t_R 22.19: 11.26** mAU^2 (0.09%); **t_R 23.01: 67.44** mAU^2 (0.51%).

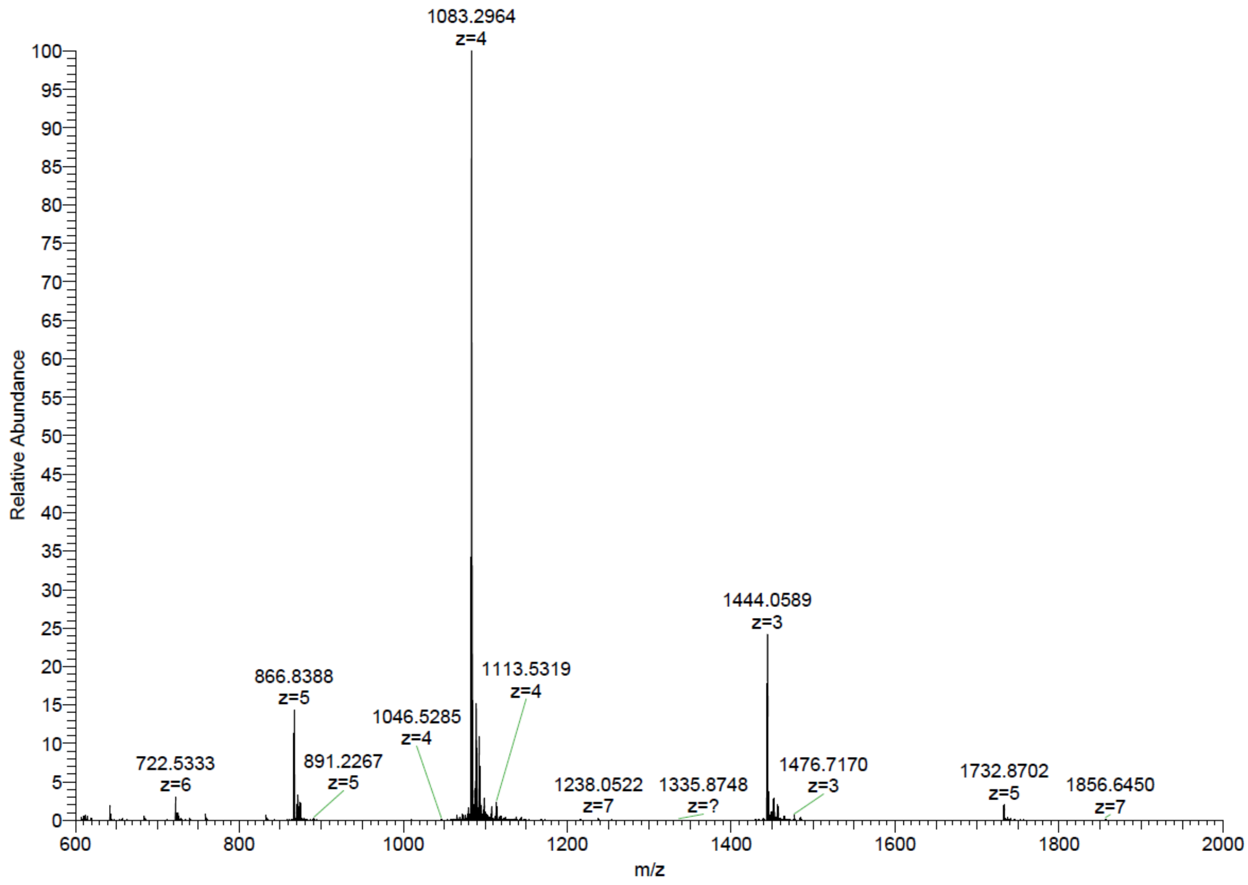

Supplementary Figure 21: Broadband mass spectrum of purified Aβ (1-40) IsoAsp23 collected by direct injection. The scan range was 600-2000 (m/z), and the population of each ion is represented by relative abundance.

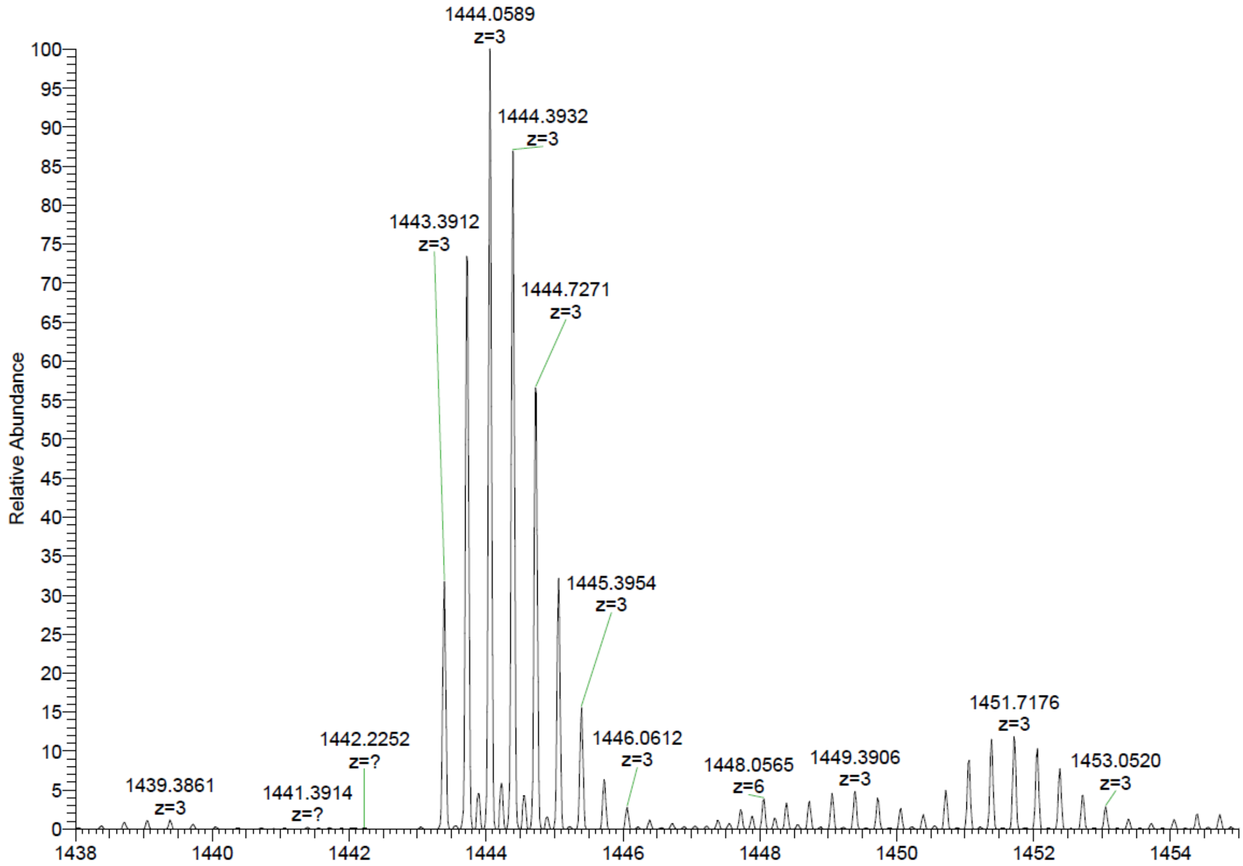

Supplementary Figure 22: Zoomed view of main Aβ (1-40) IsoAsp23 peak in broadband spectrum shown in Supplementary Figure 21. The monoisotopic $[M+3H]^{3+}$ is 1443.3912.

Supplementary References

1. Patananan, A.N., Capri, J., Whitelegge, J.P., Clarke, S.G. Non-repair Pathways for Minimizing Protein Isoaspartyl Damage in the Yeast *Saccharomyces cerevisiae*. *J. Biol. Chem*. **289**, 16936-16953 (2014).
2. Holm, L., Laasko, L. Dali server update. Nucleic acids research. 44, W351-355 (2016).
3. Garnham, C.P., Campbell, R.L., Davies, P.L. Anchored clathrate waters bind antifreeze proteins to ice. *Proc Natl Acad Sci.* **108,** 7363-7367 (2011).
4. Fitzpatrick, A.W.P et al. Cryo-EM structures of tau filaments from Alzheimer's disease. *Nature.* **547,** 185-190 (2017).
5. Li, B. et al. Cryo-EM of full-length a-synuclein reveals fibril polymorphs with a common structural kernel. *Nat Commun*. **9,** 3609 (2018).
6. Wälti, M.A. et al. Atomic-resolution structure of a disease-relevant Aβ(1-42) amyloid fibril. *Proc Natl Acad Sci USA.* **113,** E4976-4984 (2016).
7. Sgourakis, N.G., Yau, W.M., Qiang, W. Modeling an in-register, parallel "iowa" aβ fibril structure using solide-state NMR data from labeled samples with rosetta. *Structure.* **23,** 216-227 (2015).
8. Xiao, Y. et al. Aβ(1-42) fibril structure illuminates self-recognition and replication of amyloid in Alzheimer's disease. *Nat Struct Mol Biol.* **22,** 499-505 (2015).
9. Paravastu, A.K., Leapman, R.D., Yau, W.M., Tycko, R. Molecular structural basis for polymorphism in Alzheimer's beta-amyloid fibrils. *Proc Natl Acad Sci USA.* **105,** 18349-18354 (2008).
10. Lührs, T. et al. 3D structure of Alzheimer's amyloid-beta(1-42) fibrils. *Proc Natl Acad Sci USA.* **102,** 17342-17347 (2005).
11. Colvin, M.T. et al., Atomic Resolution Structure of Monomorphic Aβ42 Amyloid Fibrils. *J Am Chem Soc.* **138,** 9663-9674 (2016).
12. Gremer, L. et al. Fibril structure of amyloid-β(1-42) by cryo-electron microscopy. *Science*. **358,** 116-119 (2017).
13. Schütz, A.K. et al. Atomic-resolution three-dimensional structure of amyloid β fibrils bearing the Osaka mutation. *Angew Chem Int Ed Engl.* **54,** 331-335 (2015).
